# Direct Experimental Evidence of Biomimetic Surfaces with Chemical Modifications Interfering with Adhesive Protein Adsorption

**DOI:** 10.3390/molecules24010027

**Published:** 2018-12-21

**Authors:** Hui Yang, Wei Zhang, Ting Chen, Shizhe Huang, Baogang Quan, Min Wang, Junjie Li, Changzhi Gu, Jinben Wang

**Affiliations:** 1 CAS Key Lab of Colloid, Interface and Chemical Thermodynamics, Institute of Chemistry, Chinese Academy of Sciences, Beijing 100190, China; zhangw780@126.com (W.Z.); chenting417@iccas.ac.cn (T.C.); huangshizhe@iccas.ac.cn (S.H.); jbwang@iccas.ac.cn (J.W.); 2 Laboratory of Microfabrication, Institute of Physics, Chinese Academy of Sciences, Beijing 100190, China; quanbaogang@iphy.ac.cn (B.Q.); jjli@iphy.ac.cn (J.L.); czgu@iphy.ac.cn (C.G.); 3 Biolin Scientific (Shanghai) Trading Company Ltd., Shanghai 201203, China; Min.Wang@biolinscientific.com

**Keywords:** biomimetic surface, chemical modification, direct measurement, anti-fouling property, adhesive protein

## Abstract

Current approaches to dealing with the worldwide problem of marine biofouling are to impart chemical functionality to the surface or utilize microtopography inspired by nature. Previous reports have shown that only introducing a single method may not resist adhesion of mussels or inhibit biofouling in static forms. While it is promising to integrate two methods to develop an effective antifouling strategy, related basic research is still lacking. Here, we have fabricated engineered shark skin surfaces with different feature heights and terminated with different chemical moieties. Atomic force microscopy (AFM) with a modified colloid probe technique and quartz crystal microbalance with a dissipation n (QCM-D) monitoring method have been introduced to directly determine the interactions between adhesive proteins and functionalized surfaces. Our results indicate that the adhesion strength of probe-surface decreases with increasing feature height, and it also decreases from bare Si surface to alkyl and hydroxyl modification, which is attributed to different contact area domains and interaction mechanisms. Combining biomimetic microtopography and surface chemistry, our study provides a new perspective for designing and developing underwater anti-fouling materials.

## 1. Introduction

Marine biofouling is an intractable global problem for marine industries, ensuing serious economic cost [1,2]. In general, marine biofouling increases the fuel expenditure of seafaring vessels by up to 40% and for navy fleets the penalties that are associated with hull fouling are even higher [3,4]. Historically, toxic release was used to combat marine biofouling, however, it causes severe ecological degradation [5,6]. Since then, researchers have developed two separate strategies for anti-fouling surface modification, which are, (i) incorporating chemical functionality to the surface and (ii) creating microstructure inspired by nature [7,8,9,10]. Modulating surface chemistry can lower surface energy or form highly hydrated surface, and thereby inhibiting protein adsorption, but the coating may not be effective against "specialists" at wet adhesion, such as marine mussels [11,12]. Certain microstructures can mechanically frustrate bio-attachment under hydrodynamic conditions, but the approach may not be effective for static cases. For instance, biomimetic materials with topographical features mimicking shark skin have shown inhibition to marine biofouling at certain length scales, but it is necessary to keep the surface in constant motion [13,14]. Combining the two methods seems to be a promising way to develop novel and effective antifouling strategies [15,16], however, basic researches on modulating both surface chemistry and physical microstructure to control bioadhesion are still lacking [17].

With this in mind, we have selected mussel foot protein-1 (Mfp-1), an outer mussel anchoring protein in its byssal cuticle and rich in 3,4-dihydroxyphenylalanine (DOPA), which is considered to be responsible for good adhesive and crosslinking abilities in water [18,19,20], as a model adhesive protein, and designed biomimetic shark skin as a typical model material for self-cleaning and low adhesion surface. Our study provides direct measurements using atomic force microscopy (AFM) with a colloidal probe technique and quartz crystal microbalance with dissipation (QCM-D) monitoring on rough surfaces, and it reveals a quantitative relationship between adhesion and surface structures with different surface chemistry or feature height. We expect that our results will facilitate more theoretical studies and aid the design on anti-fouling materials.

## 2. Results and discussion

### 2.1. Fabrications and Properties of Functionalized Surfaces

**Figure 1:**
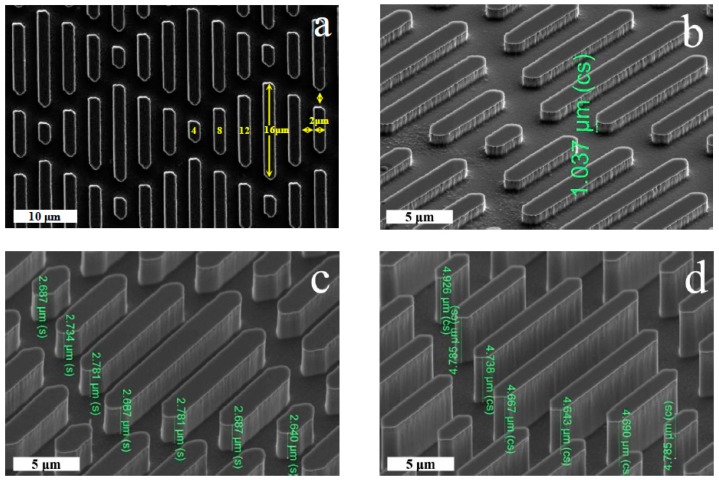

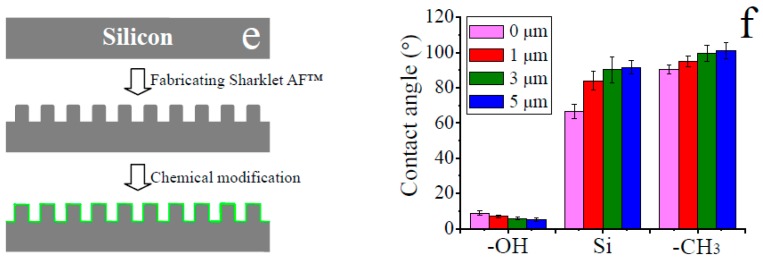
Top-down (**a**) and side-view (**b**–**d**) at 45° tilt scanning electron microscopy (SEM) images of Sharklet AF™ topography at three different feature heights: (**b**) 1 μm, (**c**) 3 μm and (**d**) 5 μm; (**e**) Schematic illustration of the fabrication procedure of the hierarchical structure; and (**f**) Water contact angle on solid surfaces with different hierarchical structures.

The surfaces with feature width and spacing of 2 μm were fabricated by photoresist coating and ICP etching (Figure 1a). It can be seen that the riblets are perpendicular to the surface and their geometric heights have a feature height of 1.0, 2.7, and 4.7 μm, respectively (Figure 1b–d), according with our Sharklet AF™ design. In order to endow the surfaces with different end-group functionalities, we modified the surfaces with hydroxyl and methyl terminated groups, as schematically illustrated in Figure 1e. The elemental composition was confirmed by XPS (Figure S1). CA of water droplet on the series of surfaces showed that it falls into the range of 5–8°, 66–91° and 90–101° for the OH–terminated surfaces, silicon surfaces, and CH_3_–terminated surfaces, respectively (Figure 1f). These results imply that the surface hydrophilicity increases with riblet height on the OH–terminated surfaces, but it decreases with feature height on the silicon and CH_3_–terminated surfaces. This can be explained by the fact that a larger surface roughness at the microscale makes a substrate more hydrophilic for the intrinsic CA below 65° and more hydrophobic for the intrinsic CA above 65° [21,22].

### 2.2. Interpretation through Direct Force Measurements

**Figure 2:**
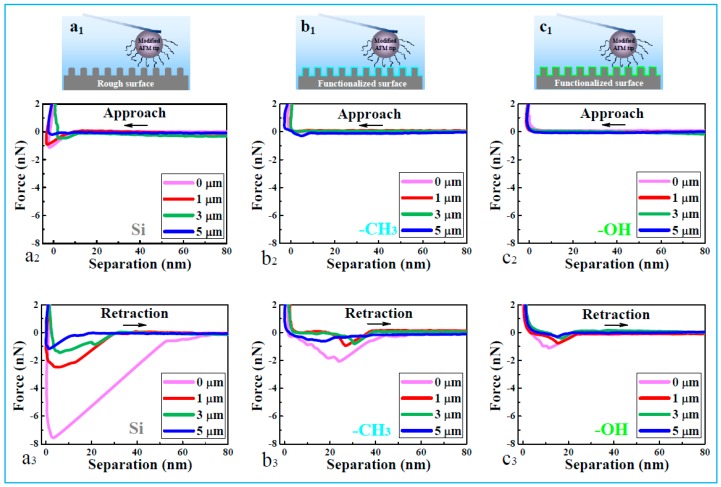
Schematic representation of approach and retraction force curves as a function of separation between 3,4-dihydroxyphenylalanine (DOPA) (modified AFM tip) and functionalized surfaces in buffer solution at different feature heights. (Bare Si surfaces: **a_1_**, **a_2_**, and **a_3_**; CH_3_-terminated surfaces: **b_1_**, **b_2_**, and **b_3_**; OH-terminated surfaces: **c_1_**, **c_2_**, and **c_3_**).

To directly determine the interactions between adhesive proteins and Sharklet AF™ surfaces, a chemically modified colloidal probe was introduced to the AFM tip. The presence of the colloid probe and the completion of chemical modification were confirmed through SEM, XPS, and CA methods (Figure 1 and Figure S2). Typical force-distance profiles are shown in Figure 2, in which positive and negative force values are denoted as repulsive and attractive interactions, respectively.

In the approach curves (Figure 2a_2_, b_2_, and c_2_), a strong and short-ranged attraction of DOPA-surface occurs only on bare Si surfaces, as compared with those on OH– and CH_3_–terminated surfaces. In the retraction curves (Figure 2a_3_, b_3_, and c_3_), the adhesion force suggests a downtrend with the increase of riblet feature height at three kinds of surfaces, and the force is much stronger in the case of Si surface than those of OH– and CH_3_–terminated surfaces at the same height.

Therefore, the adhesion strength between the AFM tip and different functionalized surfaces implies a decreasing order: Si > –CH_3_ > –OH and flat > 1 μm > 3 μm > 5 μm (Table S1). The order can be well explained by the different mechanisms of DOPA-surface interactions on the three kinds of substrates: coordination bonds occur between catechol groups of DOPA molecules and silicon surfaces, whereas hydrophobic forces dominate the adhesion between phenylalanine residues and CH_3_–terminated surfaces, and weak attractive interaction is mediated by the hydrogen bond between the hydroxyl groups of DOPA and the hydroxyl-modified surfaces [8,23]

When the feature height increases, the attractive interactions decrease for the Si surfaces, which is attributed to the reduction of contact area and thereby less coordination bond formation between DOPA and the Si surface. Further results of the surface forces are shown as histograms of adhesive forces for the series of surfaces (Figure S3). When the modified AFM tip separates from Si surfaces, the distribution of adhesive force is broad (Figure S3a–d), in comparison with those on CH_3_– and OH–terminated surfaces (Figure S3e–l). The peak value is in the range of 1.2–7.7 nN, 0.7–2.1 nN, and 0.4–1.0 nN, in the presence of Si, CH_3_–terminated, and OH–terminated surfaces (Figure S3m), respectively. With increasing feature height, the adhesive force of DOPA-surfaces becomes weak for all the three kinds of surfaces. It indicates that both the flat and rough silicon surfaces provide a strong anchoring with adhesive proteins through coordination interactions, while hydrophobic and hydrogen bond interactions are supposed to have a small contribution to the DOPA adhesion with surfaces.

### 2.3. Interpretation through In Situ Adsorption Measurements

**Figure 3:**
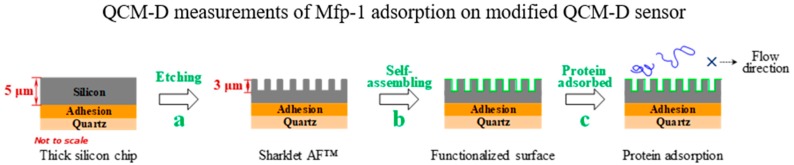

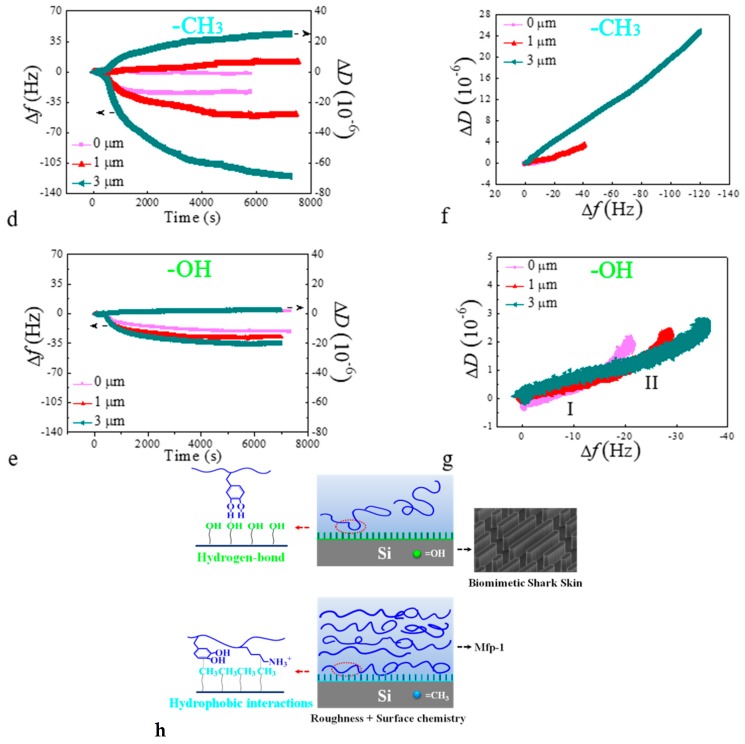
(**a**)–(**c**) Schematic illustration of the fabrication procedure of the hierarchical structure on quartz crystal microbalance with dissipation (QCM-D) sensor; Changes in frequency (Δ*f*) and dissipation (Δ*D*) as a function of time for the adsorption of Mfp-1 on the (**d**) CH_3_-terminated and (**e**) OH-terminated surfaces; Δ*D*/Δ*f* plots for the adsorption of Mfp-1 in the case of (**f**) CH_3_-terminated and (**g**) OH-terminated surfaces; and, (**h**) A schematic of adhesion mechanisms. For simplicity, only the curves of Δ*f* and Δ*D* versus time at the third overtone are shown here.

To further elucidate the protein adsorption process in hydrodynamic conditions, in situ measurements of Mfp-1 on chemically modified Sharklet AF™ surfaces were performed using the QCM-D method. Upon the addition of protein onto such kind of sensor chips, the final values of Δ*f* and Δ*D* are bigger on CH_3_–terminated surface than that on OH–terminated surface, especially in the case of feature height being 1 and 3 μm (Figure 3d,e), implying that hydroxyl modification can suppress the attachment of adhesive proteins. There is only a slight shift in dissipation of about 1 × 10^−6^ for the adsorption of Mfp-1 on OH–terminated surface, suggesting a rigid and compact layer. A clear shift of 1–20 × 10^−6^ is obtained on CH_3_–terminated surfaces, ascribed to the formation of a loose and extended layer. In the case of the hydrophilic surface, the attractive interaction is weaker than that of the hydrophobic surface (Figure 3), because the laterally tight structure of the protein-coated surface can substantially reduce the attachment of Mfp-1, leading to the lower adhesion on the hydrophilic surface. When the feature height increases, both the Δ*f* and Δ*D* values increase, taking the series of CH_3_–terminated surfaces as an example, which can be attributed to the increase of specific surface area, and therefore, there are more protein molecules that are trapped in the rough surfaces and adsorbed on the surfaces with the increasing feature height via hydrophobic interaction. The result agrees with the roughness-induced effect above the surface roughness of 3 nm, which also has been investigated through the QCM-D technique and satisfactorily explained by an advanced model [24].

Interestingly, Figure 3f shows almost linear trends of the relationship between Δ*D* and Δ*f* in the case of CH_3_–terminated surfaces at three different heights, suggesting that there are continuous increases in both values and nearly no structural rearrangements of the adsorbed protein molecules. In the case of OH–terminated surfaces (Figure 3g), the curves of Δ*D* versus Δ*f* relation reflect nonlinear trends and two different slopes *k*_I_ and *k*_II_ (as shown in Table S2), indicating that the protein adsorption processes experience two regimes. In regime I, small slopes appear since the frequency decreases rapidly, suggesting a rapid transplant and adsorption of protein molecules from solution to surfaces. In regime II, the transition point between fast and slow adsorption regime arises (Figure 3g and Figure S4), and the value of *k*_II_ increases much more than that of *k*_I_ for all cases. Therefore, a conformational change to a swelling adsorption layer can be deduced on all hydrophilic surfaces, in favor of inhibiting the adhesion of subsequent proteins to some extent when compared with that of hydrophobic surfaces with smaller slope values (Table S2) and rigid adsorption layers. Another reason for the different protein adsorption behaviors between OH-terminated and CH_3_-terminated surfaces at the same feature height can be attributed to the different interactions between protein molecules and modified surfaces. Weak hydrogen bond interactions are inferred in the case of OH-terminated surfaces and strong hydrophobic interactions are speculated in the case of CH_3_-terminated surfaces, which is in agreement with the conclusion on the adhesion mechanisms from force measurements. Furthermore, flow direction of the solution is designed along the riblets of fast-moving “shark skin”, which aids in removing weakly bound components (Figure 3h), in accordance with previous reports on the reduction of adsorbed staphylococcus aureus and algal zoospores [25,26]. When compared with Mfp-1 adsorbed on smooth surfaces with OH-SAMs, the adsorbed mass per contact surface area is reduced 20–30% on Sharklet AF™ surfaces with OH-terminals (Table S3).

Our results suggest that the micropatterned surface with chemical modification shows good antifouling property, owing to the chemical composition that suppresses protein adsorption and the removal of unstable adsorbate through the self-cleaning process on microtopographic surfaces.

## 3. Materials and Methods

### 3.1. Preparation of Surface Structures

Single-side polished n-type (100)-oriented silicon wafers were used as substrates for the microfabrication of our Sharklet AF^TM^ surface (Sharklet Technologies, Aurora, CO, USA). Silicon wafers were thoroughly cleaned with a standard RCA clean procedure, followed by the hexamethyldisilazane (HMDS) vapor prime process to increase the adhesion of photoresist on the silicon wafer surface. Thereafter, a layer of AZ 6130 photoresist (~3.5 μm in thickness) (MicroChemicals GmbH, Ulm, Germany) was applied to the silicon wafer by spin coating at 500 rpm for 5 s, followed by 3000 rpm for 40 s. Silicon wafers were then soft-baked at 100 °C for 3 min for solvent removal prior to patterning. The photoresist was in turn patterned using a Karl Suss MA6 Contact Aligner (Suss MicroTec, Garching, Germany) and photomask with a 100 mW/cm^2^ of UV exposure at 365 nm. Patterned silicon wafers were developed in AZ 300 MIF developer (H2A Technologies, Argyle, TX, USA) for 1 min. Subsequently, the photoresist pattern was transferred into silicon via a cryogenic inductively coupled plasma (ICP) reactive ion etching. The cryo-etching process was performed in an ICP etching system (Oxford Plasmalab System 100, Oxford Instruments, UK) with the optimized SF_6_/O_2_ ratio of 30/20 standard cubic centimeters per minute to get a nearly vertical etch profile (Figure 1). The Sharklet AF^TM^ (Sharklet Technologies) surface was finally obtained after a photoresist strip step in acetone.

The same structures as on the Sharklet AF^TM^ silicon surfaces were fabricated on thick SiO_2_-coated quartz crystals (5 μm, QSX999, Biolin Scientific AB, Västra Frölunda, Sweden) from Biolin Scientific AB with the same micro-fabrication process.

### 3.2. Preparation of Surface Chemistries

As-fabricated Sharklet AF^TM^ silicon wafers (1 cm × 1 cm) were cleaned using hydrofluoric acid solution for 5 min, followed by rinsing with Millipore Milli-Q grade water and nitrogen gas drying. The surfaces were treated with O_2_ plasma (Harrick, Ithaca, NY, America) for 10 min to create excess hydroxyl groups, denoted as –OH. For –CH_3_ end-groups, experiments were carried out in toluene containing 5% (*v*/*v*) n-Dodecyltrimethoxysilane (J&K Scientific, 95%) for 24 h, denoted as –CH_3_. After reaction, the surfaces were washed with ethanol and water thoroughly, followed by the confirmation of their presence via X-ray photoelectron spectroscopy (XPS), as shown in Figure S1. 

The surface structure and chemistry could be also carried out through three-dimensional (3D) printing technology in one-step process in a large scale [27,28,29], to fill in the gap between the application of ship biofouling and the structured surfaces that were proposed in this study. The surface wettability can be tuned by using water-based ink with different modified polymer component [30].

### 3.3. Surface Characterization

The morphology of the fabricated Sharklet AF^TM^ structures was examined by scanning electron microscopy (SEM, Hitachi S-5200, Hitachi High-Technologies in Europe, Krefeld, Germany). XPS was carried on Thermo Scientific ESCALab 250Xi, Thermo Fisher Scientific, Massachusetts, MA, USA with 200 W monochromated Al Kα radiation and 500 μm X-ray spot in the base pressure of 3 × 10^−10^ mbar. Contact angle (CA) measurements were done using an Attension Theta CA goniometer (Biolin Scientific, Goteborg, Sweden) at ambient temperature.

### 3.4. AFM Tip Modification and Characterization

**Figure 4:**
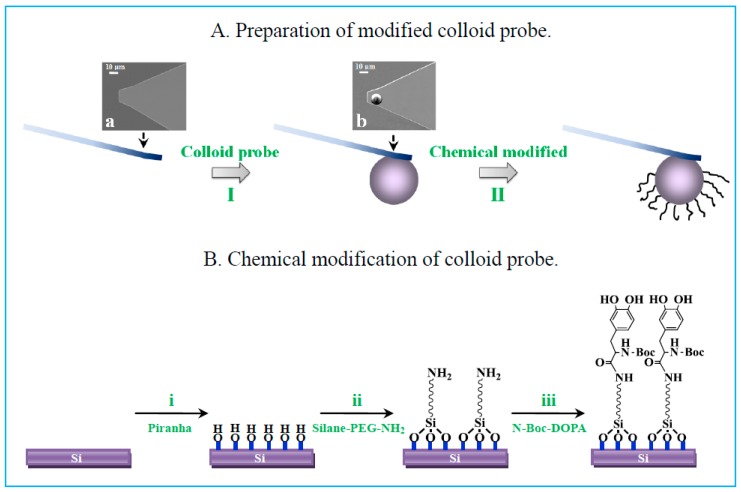
Schematic representation of chemical modification of colloidal atomic force microscopy (AFM) tip: **A**. Preparation of modified colloid probe: SEM images of (**a**) bare cantilever and (**b**) colloid probe; **B**. Chemical modification of colloid probe.

(I) Colloid probe. A silica sphere, as a colloid probe, was linked to a bare cantilever by the reformative cantilever-moving technique [31,32,33], taking such a probe shown in Figure 4 as an example. The actual spring constants of the cantilevers with the attached colloidal particles were in the range of 0.1~0.2 N·m^−1^, as determined by the “thermal tune” method [34]. Force profiles were obtained by translating the cantilever deflections (mV) and piezeotube displacements (nm) in accordance with Hooke’s law, in which the deflection sensitivity was recalibrated once the solutions were changed. (II) Chemical modification. DOPA was selected as a model adhesive protein to bind with the colloid probe. The colloid probes were cleaned in an O_2_ plasma for 15 min (reaction (i) in Figure 4), to remove impurities and form a hydroxyl layer on the colloidal surface. After rinsing with Milli-Q water, the probe was immersed into 0.5 mM silane-PEG-NH_2_ (M_W_ 3400, Nanocs, Boston, MA, USA) in toluene for a few hours, to promote the formation of an aminosilane-modified probe (reaction (ii)). The probe was then rinsed with toluene to remove unreacted reactants and was placed in an oven at 110 °C for several minutes to stabilize the silane conjugation. The probe was immersed in *N*,*N*-dimethylformamide solution with *N*-methylmorpholine, 2-(7-aza-1*H*-benzotriazole-1-yl)-1,1,3,3- tetramethyluronium hexafluorophosphate, and N-Boc-DOPA for several hours, as shown in reaction (iii). Finally, N-Boc-DOPA was end-tethered to PEG and Boc protected amine groups under to avoid electrostatic interactions [35,36]. Silicon substrates were functionalized with Boc-DOPA by the same procedure as for the probe and the resulting surfaces were characterized at each step via XPS and CA methods (Figure S2).

### 3.5. Force-Measuring Technique

The fluid chamber was full of PBS buffer containing 0.2 mM ascorbic acid at pH 7.4, to avoid the oxidation of DOPA [23,36,37]. AFM (HLCT, Bruker Corp., Santa Barbara, CA, USA) experiments were performed after equilibration for 30 min. The cantilever approached the surface at a constant speed of 1000 nm·s^−1^ and with a constant force of 3–4 nN on the surface to allow sufficient tip-surface contact.

### 3.6. Quartz Crystal Microbalance with Dissipation Monitoring (QCM-D) 

The protein adsorption processes were assessed by using a QCM-D instrument from Biolin Scientific AB (Q-sense E1, Goteborg, Sweden). Surface structure and chemistry of thick SiO_2_-coated quartz crystals at 0, 1, 3 μm feature height were created in the same way described above, with a fundamental resonant frequency of 5 MHz and a mass sensitivity constant (*C*) of 17.7 ng·cm^−2^·Hz^−1^. The QCM-D technique monitors frequency (Δ*f*) and energy dissipation (Δ*D*) of the quartz crystal oscillating shear motion at all harmonics (*n* = 1, 3, 5, ···, 13), providing information about the adsorbed mass on the surface and the viscoelastic properties of the adsorbed film. For a rigid layer, the adsorption profiles at different overtones are overlapping, and therefore, the adsorbed mass on the sensor can be obtained through the Sauerbrey equation [38] For the viscoelastic case, the layer is not fully coupled with the crystal oscillation and it undergoes a deformation under shear oscillatory motion, for which the Sauerbrey equation is not valid and the Voigt model may be employed [12,32]. In such case, the parameters, including density and viscosity of protein solutions, were adopted as 1002 kg·m^−3^ and 0.00103 Pa·s at room temperature [39]. Fitting values of shear viscosity (*η*), shear modulus (*μ*), and thickness (*h*) of adsorbed layers were obtained by modeling the experimental data using the Q-tools software package (Biolin Scientific AB, Goteborg, Sweden). All QCM-D experiments were conducted at a speed of 100 μL·min^−1^ and a room temperature of 25 °C. A baseline was established by injecting a citric acid buffer for 5 min. Mfp-1 (90%, Sigma-Aldrich Chemie Gmbh, Munich, Germany) was diluted to 100 μg·mL^−1^ in 0.1 M citric acid buffer (pH 5.5) and introduced into a fluid cell until an adsorption plateau was reached. The chips were rinsed by the buffer to detach loosely attached protein molecules thereafter and to reestablish the baseline.

## 4. Conclusions

In this study, we have fabricated biomimetic shark skin surfaces with different surface chemistries, such as hydrophilic hydroxyl –(OH) and hydrophobic methyl –(CH_3_) end groups, and studied the adhesion behaviors of adhesive proteins onto the series of surfaces through direct force-measuring and *in situ* adsorption monitoring techniques. The adhesion force of protein-surface shows a decreasing trend with the increase of riblet feature height from 1 to 5 μm, due to the reduction of contact domains. Protein adsorption under hydrodynamic conditions has been investigated with flow direction along the riblets to remove weakly bound proteins. It suggests a less favorable attachment of protein molecules from bulk solution to the OH–terminated Sharklet AF™ surface than the CH_3_–terminated one, which is in accordance with the weaker adhesive force between proteins and the former surface under static conditions. The proteins binding to the hydrophilic surface undergo structural rearrangements from rigid to soft conformation, probably leading to a slow initial stage of protein adsorption and then a swelling conformation rearrangement that can get rid of loosely adsorbed molecules. Our study provides evidence for combining surface chemistry and microtopographic patterns as an effective approach to developing anti-fouling materials suitable for the complex marine environment.

## Supplementary Materials

The following are available online: Figures S1–S4, Tables S1–S3.

## References

[1] Sedó J., Saizposeu J., Busqué F., Ruizmolina D. (2013). Catechol-based biomimetic functional materials. Adv. Mater..

[2] Anderson T.H., Yu J., Estrada A., Hammer D.M.U., Waite P.J.H., Israelachvili P.J.N. (2010). The contribution of DOPA to substrate-peptide adhesion and internal cohesion of mussel-inspired synthetic peptide films. Adv. Funct. Mater..

[3] Callow J.A., Callow M.E. (2011). Trends in the development of environmentally friendly fouling-resistant marine coatings. Nat. Commun..

[4] Epstein A.K., Hong D., Kim P., Aizenberg J. (2013). Biofilm attachment reduction on bioinspired, dynamic, micro-wrinkling surfaces. New J. Phys..

[5] Banerjee I., Pangule R.C., Kane R.S. (2011). Antifouling coatings: Recent developments in the design of surfaces that prevent fouling by proteins, bacteria, and marine organisms. Adv. Mater..

[6] Meyer B. (2003). Approaches to prevention, removal and killing of biofilms. Int. Biodeterior. Biodegr..

[7] Dalsin J.L., Hu B.H., Lee B.P., Messersmith P.B. (2003). Mussel adhesive protein mimetic polymers for the preparation of nonfouling surfaces. J. Am. Chem. Soc..

[8] Chen T., Yang H., Gao H., Fu M., Huang S., Zhang W., Hu G., Liu F., Ma A., Sun K. (2017). Adsorption and orientation of 3,4-dihydroxy-L-phenylalanine onto tunable monolayer films. J. Phys. Chem. C.

[9] Nishimoto S., Bhushan B. (2013). Bioinspired self-cleaning surfaces with superhydrophobicity, superoleophobicity, and superhydrophilicity. RSC Adv..

[10] Krishnan S., Wang N., Ober C.K., Finlay J.A., Callow M.E., Callow J.A., Hexemer A., Sohn K.E., Kramer E.J., Fischer D.A. (2006). Comparison of the fouling release properties of hydrophobic fluorinated and hydrophilic PEGylated block copolymer surfaces: Attachment strength of the diatom Navicula and the green alga Ulva. Biomacromolecules.

[11] Prime K.L., Whitesides G.M. (1991). Self-assembled organic monolayers: Model systems for studying adsorption of proteins at surfaces. Science.

[12] Hook F., Kasemo B., Nylander T., Fant C., Kristin Sott A., Elwing H. (2001). Variations in coupled water, viscoelastic properties, and film thickness of a mefp-1 protein film during adsorption and cross-linking:  A quartz crystal microbalance with dissipation monitoring, ellipsometry, and surface plasmon resonance study. Anal. Chem..

[13] Schumacher J.F., Carman M.L., Estes T.G., Feinberg A.W., Wilson L.H., Callow M.E., Callow J.A., Finlay J.A., Brennan A.B. (2007). Engineered antifouling microtopographies—effect of feature size, geometry, and roughness on settlement of zoospores of the green alga Ulva. Biofouling.

[14] Ralston E., Swain G. (2009). Bioinspiration—the solution for biofouling control?. Bioinspir. Biomim..

[15] Liu H., Ding Y., Ao Z., Zhou Y., Wang S., Jiang L. (2015). Fabricating surfaces with tunable wettability and adhesion by ionic liquids in a wide range. Small.

[16] Baxamusa S.H., Gleason K.K. (2009). Random copolymer films with molecular-scale compositional heterogeneities that interfere with protein adsorption. Adv. Funct. Mater..

[17] Vladkova T. (2009). Surface modification approach to control biofouling. Mar. Ind. Biofoul..

[18] Lu Q., Danner E., Waite J.H., Israelachvili J.N., Zeng H., Hwang D.S. (2013). Adhesion of mussel foot proteins to different substrate surfaces. J. R. Soc. Interface.

[19] Holtenandersen N., Zhao H., Waite J.H. (2009). Stiff coatings on compliant biofibers: The cuticle of mytilus californianus byssal threads. Biochemistry.

[20] Danielj R., Ali M., Herbertj W. (2010). Diverse strategies of protein sclerotization in marine invertebrates: Structure-property relationships in natural biomaterials. Adv. Insect Physiol..

[21] Tian Y., Jiang L. (2013). Wetting: Intrinsically robust hydrophobicity. Nat. Mater..

[22] Wu X., Liu M., Zhong X., Liu G., Wyman I., Wang Z., Wu Y., Yang H., Wang J. (2017). Smooth water-based antismudge coatings for various substrates. ACS Sustain. Chem. Eng..

[23] Zhang W., Yang H., Liu F., Chen T., Hu G., Guo D., Hou Q., Wu X., Su Y., Wang J. (2017). Molecular interactions between DOPA and surfaces with different functional groups: A chemical force microscopy study. RSC Adv..

[24] Rechendorff K., Hovgaard M.B., Foss M., Besenbacher F. (2007). Influence of surface roughness on quartz crystal microbalance measurements in liquids. J. Appl. Phys..

[25] Chung K., Schumacher J., Sampson E., Burne R., Antonelli P., Brennan A. (1992). Impact of engineered surface microtopography on biofilm formation of Staphylococcus aureus. Langmuir.

[26] Schumacher J.F., Aldred N., Callow M.E., Finlay J.A., Callow J.A., Clare A.S., Brennan A.B. (2007). Species-specific engineered antifouling topographies: Correlations between the settlement of algal zoospores and barnacle cyprids. Biofouling.

[27] Gopinathan J., Noh I. (2018). Recent trends in bioinks for 3D printing. Biomaterials Research.

[28] Rebeiz G.M., Muldavin J.B., Schoenlinner B., Tan G.L. (2004). RF MEMS: Theory, Design, and Technology.

[29] Chang J., He J., Lei Q., Li D. (2018). Electrohydrodynamic printing of microscale PEDOT: PSS-PEO features with tunable conductive/thermal properties. ACS Appl. Mater. Interfaces.

[30] Ramirez J.C.C., Tumolva T.P. (2018). Analysis and optimization of water-based printing ink formulations for polyethylene films. Appl. Adhes. Sci..

[31] Yu D., Yang H., Wang H., Cui Y., Yang G., Zhang J., Wang J. (2014). Interactions between colloidal particles in the presence of an ultrahighly charged amphiphilic polyelectrolyte. Langmuir.

[32] Yang H., Duan H., Wu X., Wang M., Chen T., Liu F., Huang S., Zhang W., Chen G., Yu D. (2016). Self-assembly behavior of ultrahighly charged amphiphilic polyelectrolyte on solid surfaces. Langmuir.

[33] Zhang W., Su Y., Liu F., Yang H., Wang J. (2017). Study of interactions between 3,4-dihydroxyphenylalanine and surfaces with nano-, micro-and hierarchical structures using colloidal probe technology. Acta Physico-Chim. Sin..

[34] Hutter J.L., Bechhoefer J. (1993). Calibration of atomic-force microscope tips. Rev. Sci. Instrum..

[35] Lee H., Scherer N.F., Messersmith P.B. (2006). Single-molecule mechanics of mussel adhesion. Proc. Natl. Acad. Sci. USA.

[36] Li Y., Qin M., Li Y., Cao Y., Wang W. (2014). Single molecule evidence for the adaptive binding of DOPA to different wet surfaces. Langmuir.

[37] Yu J., Wei W., Danner E., Israelachvili J.N., Waite J.H. (2011). Effects of interfacial redox in mussel adhesive protein films on mica. Adv. Mater..

[38] Sauerbrey G. (1959). Use of vibrating quartz for thin film weighing and microweighing. Eur. Phys. J. A.

[39] Huang S., Hou Q., Guo D., Yang H., Chen T., Liu F., Hu G., Zhang M., Zhang J., Wang J. (2017). Adsorption mechanism of mussel-derived adhesive proteins onto various self-assembled monolayers. RSC Adv..

