# Supplementary material for: Direct Experimental Evidence of Biomimetic Surfaces with Chemical Modifications Interfering with Adhesive Protein Adsorption
Source: Molecules. 2018 Dec 21;24(1):27. doi: 10.3390/molecules24010027 (PMC6337514; doi:10.3390/molecules24010027)
Supplement: Supplementary file 1 [file molecules-24-00027-s001.pdf]

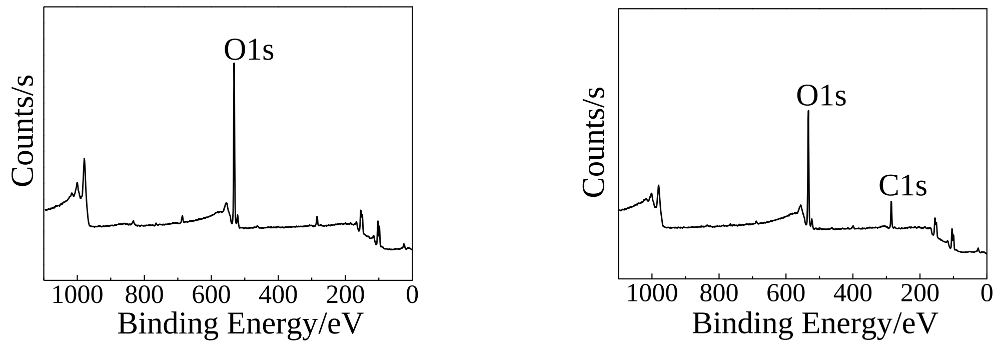

(a) OH-terminated

(b) CH_3-terminated

Figure S1. XPS spectra of (a) OH- and (b) CH_3-terminated Sharklet AF™ surfaces.

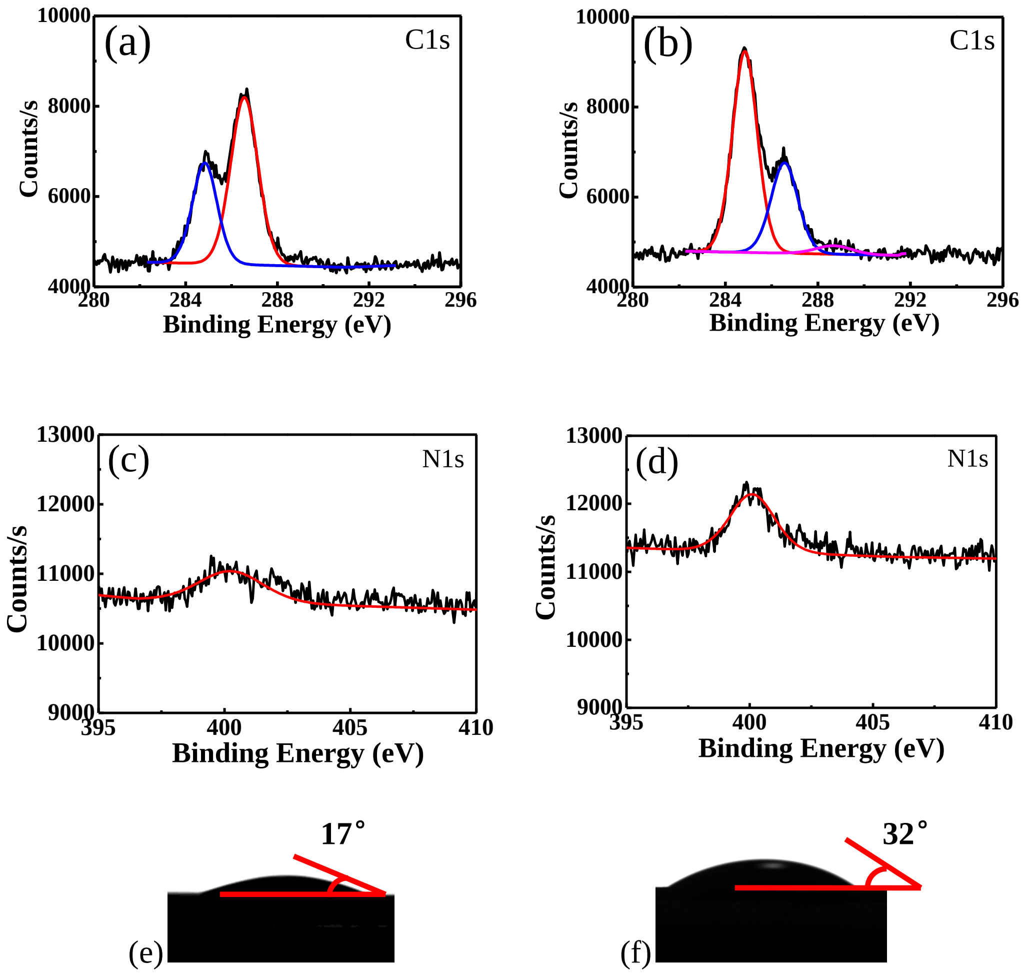

Figure S2. XPS C1s (a, b), N1s (c, d) spectra, and contact angle (e, f) of NH_2−terminated (left) and DOPA−terminated (right) surfaces, respectively.

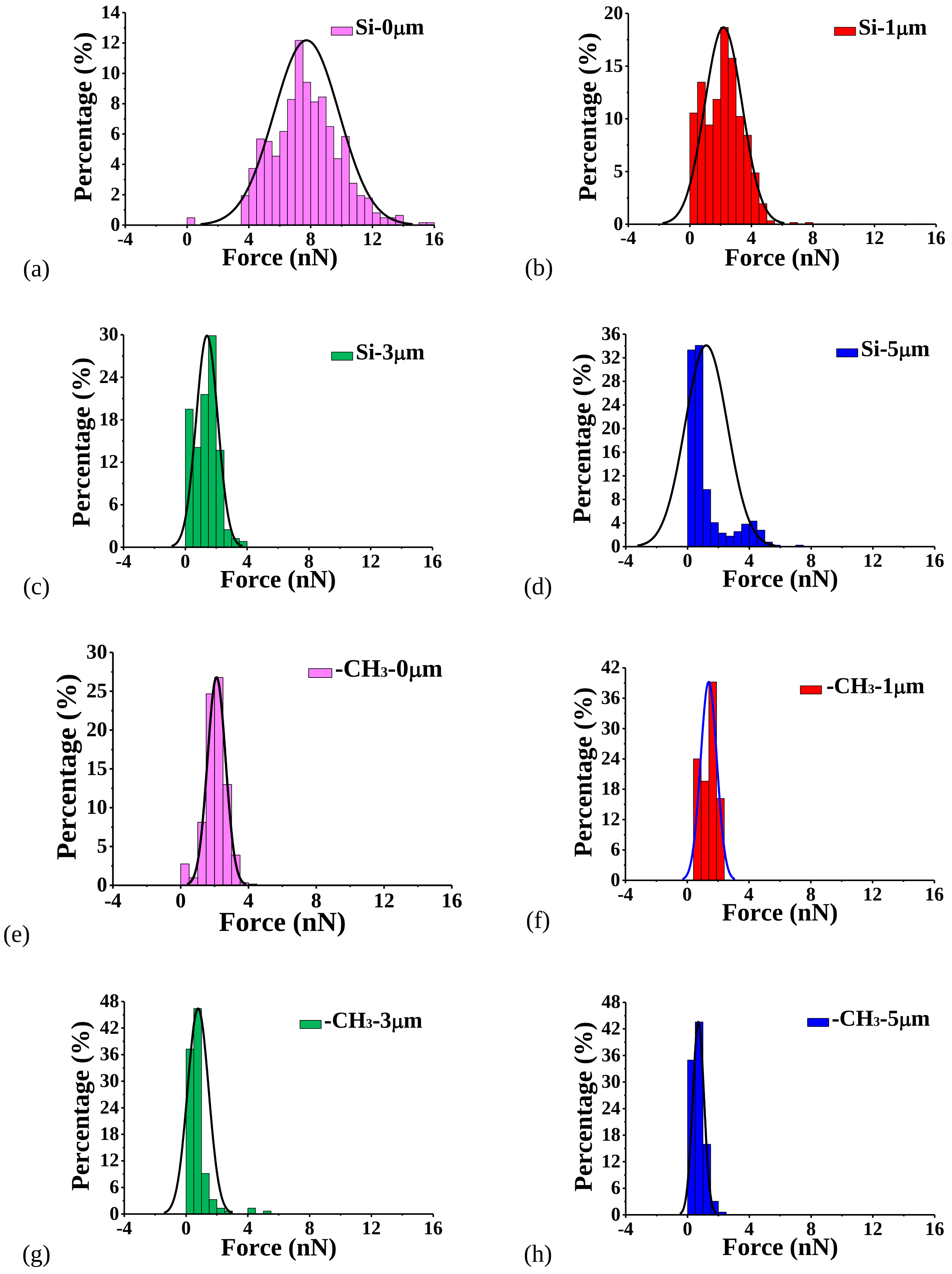

(a)
Si-0μm
Percentage (%)
Force (nN)
(b)
Si-1μm
Percentage (%)
Force (nN)
(c)
Si-3μm
Percentage (%)
Force (nN)
(d)
Si-5μm
Percentage (%)
Force (nN)
(e)
-CH3-0μm
Percentage (%)
Force (nN)
(f)
-CH3-1μm
Percentage (%)
Force (nN)
(g)
-CH3-3μm
Percentage (%)
Force (nN)
(h)
-CH3-5μm
Percentage (%)
Force (nN)

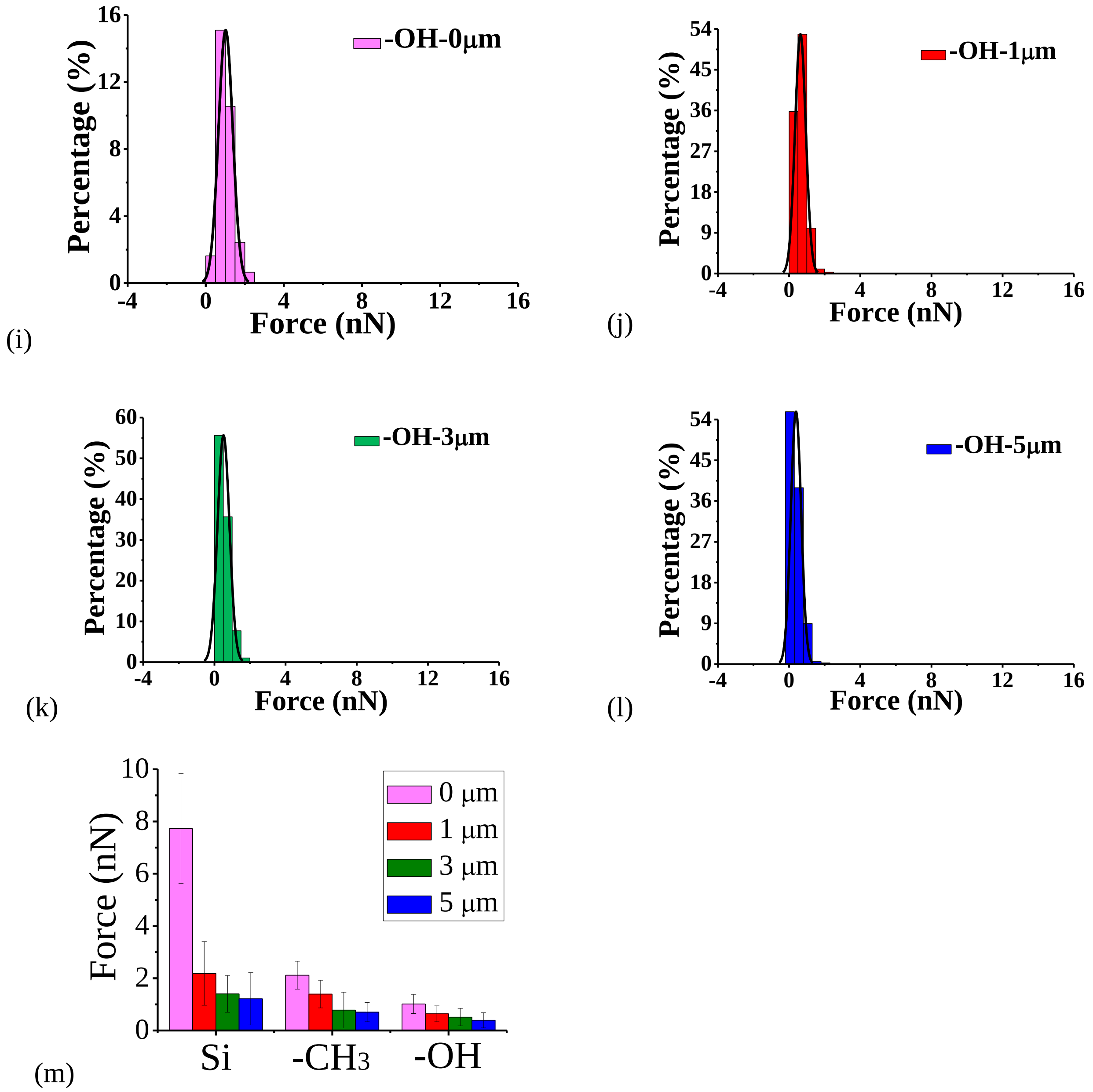

Figure S3. Histograms of adhesion forces in the presence of (a)~(d) bare Si surfaces, (e)~(h) CH_3-terminated surfaces, and (i)~(l) OH-terminated surfaces, all at different feature heights. Black lines correspond to a Gaussian fit. (m) Comparison of all the average adhesive forces.

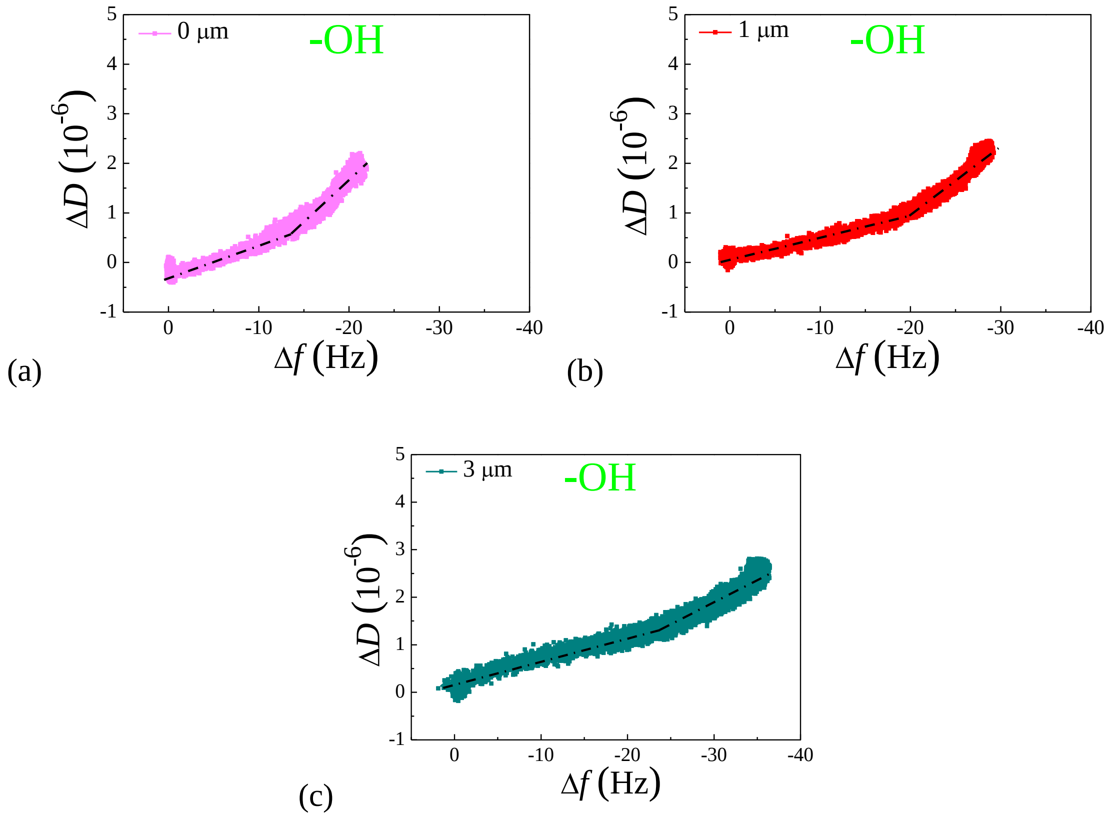

Figure S4 Changes in frequency (Δ*f*) and dissipation (Δ*D*) as a function of time for the adsorption of Mfp-1 on OH-terminated surfaces at the feature height of (a) 0 μm, (b) 1 μm, and (c) 3 μm.

Table S1. Average adhesive forces in the presence of different surfaces.

		Average adhesive force (nN)		
Different surfaces		Si	$-CH_3$	-OH
Height (μm)	0	7.7±1.0	2.1±0.4	1.0±0.3
	1	2.2±1.3	1.4±0.5	0.6±0.3
	3	1.4±1.2	0.8±0.5	0.5±0.2
	5	1.2±0.7	0.7±0.4	0.4±0.2

Table S2. Values of k_I and k_{II} for the first and second kinetic processes on OH-terminated surfaces and k values for the CH_3-terminated surfaces.

	OH-terminated surfaces		CH_3-terminated surfaces
Different height (μm)	k_I (-10^{-8}/Hz)	k_{II} (-10^{-8}/Hz)	k (-10^{-8}/Hz)
0	0.24	1.87	0.06
1	0.09	1.62	0.09
3	0.11	0.68	0.21

Table S3. Comparison of adsorbed masses on smooth and rough surfaces.

Different surfaces		Adsorbed mass (ng/cm^2)	Contact area (mm^2)	Average adsorbed mass (ng/cm^2)
Smooth surface	OH–termination	372.0	78.5	372
	CH_3–termination	624.1	78.5	624
Rough surface with 1 μm in height	OH–termination	455.2	117.8	303
	CH_3–termination	1564.3	117.8	1042
Rough surface with 3 μm in height	OH–termination	653.2	196.3	261
	CH_3–termination	3081.8	196.3	1232
